# Factors associated with having a steady homosexual partner among men who have sex with men, based on internet recruitment

**DOI:** 10.3389/fpubh.2025.1508952

**Published:** 2025-01-22

**Authors:** Peng Zhang, Weiyong Chen, Jing Li, Yun Fu, Fang Wang, Xing Wang, Zhongrong Yang

**Affiliations:** ^1^Huzhou Center for Disease Control and Prevention, Huzhou, Zhejiang, China; ^2^Department of HIV/STD Control and Prevention, Zhejiang Provincial Center for Disease Control and Prevention, Hangzhou, Zhejiang, China; ^3^Huzhou Third Municipal Hospital, The Affiliated Hospital of Huzhou University, Huzhou, Zhejiang, China

**Keywords:** internet, men who have sex with men, HIV, infection, homosexual

## Abstract

**Objective:**

This study analyzed the status and factors associated with having a steady homosexual partner (HP) among men who have sex with men (MSM) to provide a scientific basis for developing prevention and control strategies for acquired immunodeficiency syndrome in this population.

**Methods:**

A questionnaire survey was conducted among study participants who were MSM recruited online by a non-governmental organization in May 2024. The participants were divided into two groups based on whether they had a steady HP. Univariate and multivariate logistic regression analyses were used to evaluate the factors influencing whether the participants had a steady HP.

**Results:**

We surveyed 604 participants;211 (34.93%) had a current steady HP. Multivariate logistic regression analysis showed that older participants having a steady HP (adjusted odds ratio [aOR]: 1.04; 95% confidence interval [CI]: 1.01–1.07)was associated with an average monthly income >6,000 Chinese yuan (aOR: 1.47; 95% CI: 1.01–2.16), a belief that using enhancers such as Rush Popper increases the human immunodeficiency virus (HIV) infection risk (aOR: 1.56; 95% CI: 1.02–2.41), identification as purely homosexual (aOR: 2.02; 95% CI: 1.37–2.99), having engaged in anal sex with a HP in the previous 6 months (aOR: 4.13; 95% CI:2.51–6.90), and knowing the HIV status of their HP (aOR: 1.88; 95% CI: 1.17–3.06). Participants less likely to have a steady HP had used condoms consistently during anal sex with a HP in the prior 6 months (aOR: 0.56; 95% CI: 0.35–0.90) and engaged in one-night stands with HPs (aOR: 0.44; 95% CI: 0.29–0.65).

**Conclusion:**

A low proportion of participants had steady HPs (34.93%). Therefore, public education for MSM should be promoted to encourage individuals to improve their awareness of HIV infection risks. Sexual responsibility, including correct use and risks of enhancers like Rush Popper, and avoiding high-risk behaviors, such as one-night stands, is crucial to reducing the risks of HIV and sexually transmitted infections.

## 1 Introduction

Acquired immunodeficiency syndrome (AIDS) is a severe infectious disease resulting from human immunodeficiency virus (HIV) infection (1, 2). HIV compromises the body’s immune system, rendering patients more vulnerable to various diseases with accelerated disease progression, culminating in AIDS (3). Despite substantial advancements in HIV prevention and treatment strategies in recent decades, AIDS remains a significant global health challenge (2, 4, 5). Men who have sex with men (MSM) are a distinct group with unique sociosexual backgrounds and identity challenges (6, 7). The HIV prevalence rate among this group significantly surpasses that of other demographics, causing substantial health complications and societal repercussions (8). Efforts aimed at HIV prevention and control within the MSM community have encountered multiple hurdles, including cultural barriers, societal prejudices, and policy constraints, necessitating a collaborative societal approach to fortify preventive and intervention measures (9).

MSM are crucial in public health because their specific anal sexual practices can result in the transmission of sexually transmitted infections (STDs), including HIV/AIDS (10, 11), emphasizing the need for an investigation of sexual partner–selection behaviors. Compared to casual sexual partners, stable partner relationships typically present a lower risk of HIV transmission (12). The communication and trust inherent in stable partnerships encourage both individuals to engage in safer sexual behaviors, thereby reducing the likelihood of HIV infection. Furthermore, stable relationships offer emotional support and social recognition, which significantly influence health-related behavioral choices. Research indicates that MSM in stable partnerships demonstrate more favorable practices regarding safe sexual behaviors, including higher frequencies of condom use and lower rates of substance abuse (13, 14). Understanding the characteristics and influential factors of stable partnerships can serve as a foundation for developing more effective prevention and intervention strategies aimed at reducing HIV transmission. Research on partner selection behaviors among MSM suggests that diverse background factors influence their choice of sexual partners, including age, economic status, self-identified sexual orientation, sexual preferences, number of partners, and sexual health awareness (15–17). A comprehensive analysis of these factors can provide insights into the prevalence of stable homosexual partnerships (HP) within the MSM community and facilitate the prediction of future trends. Understanding these factors is pivotal for devising effective strategies for promoting sexual health, mitigating the risk of STDs, and enhancing the overall social health status of MSM (18).

Recruiting MSM through online research platforms is common (16, 19, 20). However, studies examining stable HPs and their related factors are limited for this demographic population. This study seeks to bridge this research gap by investigating the factors underlying the characteristics of the MSM population and examining their social interactions and sexual behaviors. The objective of this study is to analyze the status and factors associated with having a steady HP among MSM. Based on the research findings, health education and intervention measures tailored for the MSM community will be developed to enhance awareness of safe sexual practices and reduce the risk of HIV infection.

## 2 Materials and methods

### 2.1 Ethical statement

The research protocol was approved by the Ethics Committee of the Huzhou City Center for Disease Control and Prevention (Approval No.: HZ2023003). The participants provided their written informed consent to participate in this study.

### 2.2 Study design and participants

This cross-sectional survey study was conducted by a non-governmental organization (NGO) named Ma’anqianxun health information consultation center in Zhejiang province in May 2024 by recruiting MSM online and administering a questionnaire. The participants were MSM recruited online who initially completed the questionnaire through WeChat, the age range of participants is between 18 and 60 years old. MSM refers to have engaged in sexual activities between males, including masturbation, oral sex, and anal sex.

The questionnaire was set up on the Wenjuanxing platform, allowing each WeChat ID to complete it once. Effective quality control measures were implemented through questionnaire restrictions, including mandatory questions, logic jumps, and response range limitations, to obtain valid questionnaires that met the recruitment criteria. The survey staff received standardized training for using a uniform questionnaire for data collection. Before the survey, the investigators explained the purpose, significance, methodology, and privacy protection policy to the participants; this information was included at the beginning of the questionnaire. Participants were informed that the survey aimed to develop prevention strategies for HIV and sexually transmitted infections among MSM, that the survey would be anonymous, and that only group data, not personal data, would be analyzed.

This study utilized internet recruitment rather than random sampling, thereby we employed a non-probability sampling method akin to snowball sampling. To determine the sample size, the following calculation method was applied: utilizing data from behavioral monitoring obtained in repeated surveys, we derived the formula N = 400 × Q / P. Here, P represents the estimated proportion of the relevant behavior occurring during the survey; specifically, it is based on the proportion of MSM who reported unprotected anal intercourse, estimated between 28.7 and 53.0% according to related surveys (21–23). In this study, we used *p* = 47.5%, Q = 1 - P. Given these parameters, the study necessitates a minimum of 442 participants recruited via the internet.

The inclusion criteria were age ≥ 18 years, have engaged in sexual activities between males (including masturbation, oral sex, and anal sex), and willingness to participate in the survey after providing informed consent. We excluded participants who were aged <18 years, who recruited sexual partners offline or at fixed locations, who were unwilling to participate after having provided informed consent, or had mental or cognitive disorders based on self-reporting.

We investigated a total of 626 participants. Among 626 initial participants, 604 provided informed consent before completing the questionnaire; 22 individuals who did not consent to participate were excluded.

### 2.3 Survey content

Our questionnaire design was mainly based on the Chinese MSM sentinel monitoring questionnaire and the college student survey questionnaire (24–26). After conducting a pre survey and improving the questionnaire variable settings, we conducted a formal questionnaire survey. The content of survey questionnaire included general demographic characteristics (e.g., age, educational level, average monthly income), sexual behavior–related characteristics attitudes toward sex, condom use during sexual activities, status of steady sexual partners, sexual activity in the previous 6 months, alcohol or drug use during homosexual sexual activities, receipt of HIV prevention services in the prior year (e.g., distribution of informational materials and condoms, counseling services, training workshops), HIV testing in the previous year, self-efficacy in condom use, and awareness of the HIV infection status of HPs.

### 2.4 Definition of related indicators

A steady partner was defined by the participant as a sexual partner who met on a regular basis, or when the participant identified the other man to be his primary/steady partner (27). Commercial sexual behavior refers to sexual activities involving monetary transactions, including sugar-daddy arrangements, prostitution, and solicitation. The receipt of prevention services refers to whether a participant received HIV/AIDS-related preventive services in the previous year, including the distribution of informational materials and condoms, counseling services, and training workshops. A one-night stand refers to a temporary sexual encounter between MSM. Condom use self-efficacy is measured by a scale developed by Hanna et al. (28), consisting of three main questions: confidence in discussing condom use with sexual partners before engaging in sexual activity, lack of confidence in abstaining from sex if a partner refuses to use or brings a condom, and confidence in preparing condoms in advance before sexual activity. Each question is scored for five response options: very confident (3 points), confident (2 points), somewhat confident (1 point), not confident (0 points), and not at all confident (−1 point). Total scores were divided into groups of 9, 5–8, and < 4 points. Cronbach’s alpha coefficient for this measurement was 0.816.

### 2.5 Statistical analysis

Statistical analyses were performed using R 4.4.1. Descriptive statistics were used for continuous data (mean ± standard deviation); categorical data were analyzed as frequencies or percentages using the chi-square test. Participants were grouped based on whether they currently have a steady HP as the dependent variable (1 = yes, 0 = no). Variables with *p* < 0.1 in the univariate regression analysis were included as independent variables in the multivariate logistic regression model using the Enter method. Significance was set at *p* < 0.05.

## 3 Results

### 3.1 General demographic characteristics

The study included 604 participants, with an average age of 28.04 ± 6.43 years (range 19–53). Rural household registrations accounted for 38.58% (233/604) of the participants. Those with a monthly income >6,000 Chinese yuan (CNY) comprised 41.39% (250/604). Individuals with at least a college degree accounted for 77.48% (468/604) of participants. Participants who reported harmonious family relationships constituted 54.80% (331/604) of the sample. Students accounted for 20.53% (124/604) of participants. Table 1 provides these results.

**Table 1:** Analysis of factors associated with having a steady homosexual partner.

Variables	Whether currently have a steady homosexual partner				Univariate analysis		Multivariate analysis	
	Yes (*n* = 211)		No (*n* = 393)		OR (95%CI)	*p*	aOR (95%CI)	*p*
	*n*	%	*n*	%				
Age (mean ± SD, yrs)	28.75 ± 7.06		27.69 ± 6.06		1.03 (1.00–1.05)	0.054	1.04 (1.01–1.07)	0.006
Residence registration								
Urban areas	126	34.0	245	66.0	Ref.			
Rural areas	85	36.5	148	63.5	1.12 (0.79–1.57)	0.528		
Educational level								
High school or below	46	33.8	90	66.2	Ref.			
College degree or above	165	35.3	303	64.7	1.07 (0.71–1.60)	0.757		
Student								
No	173	36.0	307	64.0	Ref.			
Yes	38	30.6	86	69.4	0.78 (0.51–1.19)	0.262		
Average monthly income (CNY^*^)								
≤6,000	109	30.8	245	69.2	Ref.		Ref.	
>6,000	102	40.8	148	59.2	1.55 (1.10–2.17)	0.011	1.47 (1.01–2.16)	0.046
Family relationships								
General/Disharmonious	85	31.1	188	68.9	Ref.		Ref.	
Harmonious	126	38.1	205	61.9	1.36 (0.97–1.91)	0.076	1.31 (0.90–1.91)	0.165
Whether think that “anal sex with men” is more likely to be infected with HIV than “vaginal sex with women, anal sex with women, or oral sex with men”								
No	21	25.6	61	74.4	Ref.		Ref.	
Yes	190	36.4	332	63.6	1.66 (1.00–2.87)	0.059	1.21 (0.68–2.23)	0.523
Whether think using enhancers like Rush Popper increases the risk of HIV infection								
No	50	26.6	138	73.4	Ref.		Ref.	
Yes	161	38.7	255	61.3	1.74 (1.19–2.56)	0.004	1.56 (1.02–2.41)	0.043
Whether received HIV/AIDS prevention services (including promotional materials and distribution of condoms, counseling services, training sessions, etc.) in the past year								
No	60	30.0	140	70.0	Ref.		Ref.	
Yes	151	37.4	253	62.6	1.39 (0.97–2.01)	0.074	1.04 (0.68–1.59)	0.849
Whether consider self sexual orientation to be purely homosexual								
No	71	26.4	198	73.6	Ref.		Ref.	
Yes	140	41.8	195	58.2	2.00 (1.42–2.84)	<0.001	2.02 (1.37–2.99)	<0.001
Whether have anal sex with the homosexual partner in the last 6 months								
No	53	21.4	195	78.6	Ref.		Ref.	
Yes	158	44.4	198	55.6	2.93 (2.04–4.27)	<0.001	4.13 (2.51–6.90)	<0.001
Whether consistently used condoms during anal intercourse with homosexual partners in the last 6 months								
No	129	32.3	271	67.8	Ref.		Ref.	
Yes	82	40.2	122	59.8	1.41 (0.99–2.00)	0.053	0.56 (0.35–0.90)	0.017
Whether engaged in alcohol consumption, drug use, or the use of sexual enhancement drugs (such as Viagra) when engaging in sexual activity with homosexual partners								
No	175	33.8	343	66.2	Ref.			
Yes	36	41.9	50	58.1	1.41 (0.88–2.24)	0.147		
Whether have ever engaged in one-night stands of homosexual activity								
No	127	38.0	207	62.0	Ref.		Ref.	
Yes	84	31.1	186	68.9	0.74 (0.52–1.03)	0.077	0.44 (0.29–0.65)	<0.001
Whether engaged in sexual activity with the heterosexual partner in the past 6 months								
No	186	34.3	357	65.7	Ref.			
Yes	25	41.0	36	59.0	1.33 (0.77–2.28)	0.297		
Condom use self-efficacy measurement (Scores)								
4 or below	46	26.4	128	73.6	Ref.		Ref.	
5–8	66	33.8	129	66.2	1.42 (0.91–2.24)	0.123	1.11 (0.66–1.85)	0.694
9	99	42.1	136	57.9	2.02 (1.33–3.12)	0.001	1.50 (0.91–2.51)	0.116
Whether want to know the HIV infection status of homosexual partner								
No	15	22.4	52	77.6	Ref.		Ref.	
Yes	196	36.5	341	63.5	1.99 (1.12–3.75)	0.025	0.79 (0.37–1.71)	0.537
Whether aware of the HIV infection status of homosexual partner								
No	44	22.9	148	77.1	Ref.		Ref.	
Yes	167	40.5	245	59.5	2.29 (1.56–3.41)	<0.001	1.88 (1.17–3.06)	0.01
Whether worried about getting HIV from homosexual partner								
No	80	38.1	130	61.9	Ref.			
Yes	131	33.2	263	66.8	0.81 (0.57–1.15)	0.234		
Whether believe that condoms can effectively prevent the transmission of HIV during homosexual encounters								
No	15	19.7	61	80.3	Ref.		Ref.	
Yes	196	37.1	332	62.9	2.40 (1.36–4.49)	0.004	1.78 (0.90–3.66)	0.106
Whether had been tested for HIV in the past year								
No	25	21.0	94	79.0	Ref.		Ref.	
Yes	186	38.4	299	61.6	2.33 (1.47–3.84)	<0.001	1.39 (0.80–2.47)	0.244
Whether believe that taking specific medications regularly before engaging in high-risk sexual activity (such as pre-exposure prophylaxis for HIV) can reduce the risk of HIV infection								
No	28	24.6	86	75.4	Ref.		Ref.	
Yes	183	37.3	307	62.7	1.83 (1.16–2.95)	0.011	1.03 (0.60–1.81)	0.906

^*^CNY, Chinese yuan.

### 3.2 Factors associated with having steady homosexual partners

Among the participants, 211 (34.93%, 211/604) had HPs. The variables with *p* < 0.1 in the univariate analysis included age, average monthly income, family relationships, a belief that “anal sex with men” is more likely to transmit HIV than “vaginal sex with women, anal sex with women, or oral sex with men,” a belief that using enhancers such as Rush Popper increases the risk of HIV infection, receipt of HIV prevention services in the past year, self-identified sexual orientation as purely homosexual, engagement in anal sex with HPs in the previous 6 months, consistent condom use during anal sex with HPs in the prior 6 months, occurrence of one-night stands with HPs, self-efficacy in condom use, desire to know the HIV infection status of HPs, awareness of the HIV infection status of HPs, belief that condoms prevent HIV transmission among HPs, HIV testing in the prior year, and willingness to minimize HIV risk through specific medications (including pre-exposure prophylaxis for HIV) before engaging in high-risk sexual activities.

**Figure 1:**
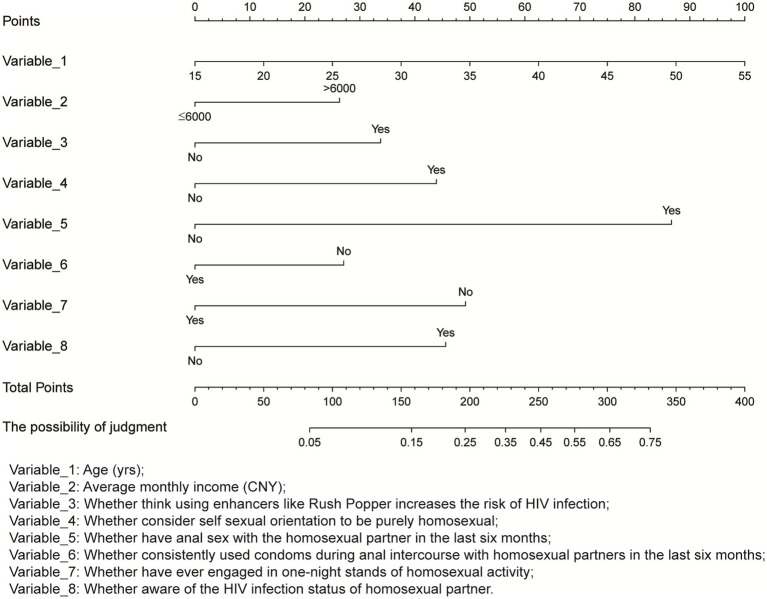
Nomogram of factors associated with having steady homosexual partner among participants.

Variables with *p* < 0.1 in the univariate analysis were included in the multivariate logistic regression analysis. The analysis indicated (Table 1) that each yearly increase in age increased the likelihood of participants having a steady HP by 4% (adjusted odds ratio [aOR]: 1.04; 95% confidence interval [CI]: 1.01–1.07). Participants with an average monthly income exceeding 6,000 CNY were 47% more likely to have a steady HP (aOR, 1.47; 95% CI: 1.01–2.16). Individuals who believed that using enhancers such as Rush Popper increased the risk of HIV infection had a 56% greater likelihood of having a steady HP (aOR: 1.56; 95% CI: 1.02–2.41). Participants who self-identified as purely homosexual were 102% more likely to have a steady HP (aOR: 2.02; 95% CI: 1.37–2.99). Those who engaged in anal sex with a HP in the previous 6 months were 313% more likely to have a steady HP (aOR: 4.13; 95% CI: 2.51–6.90). Participants aware of their HIV infection status had an 88% greater likelihood of having a steady HP (aOR: 1.88; 95% CI: 1.17–3.06). Individuals who used condoms consistently during anal sex with HPs in the prior 6 months were 44% less likely to have a steady HP (aOR: 0.56; 95% CI: 0.35–0.90). Those who engaged in one-night stands with HPs were 56% less likely to have a steady HP (aOR 0.44, 95% CI: 0.29–0.65). Nomogram of factors associated with having steady homosexual partner among participants is shown in Figure 1.

## 4 Discussion

MSM area high-risk group for HIV because of factors that often result in reluctance to undergo HIV testing or access HIV-related prevention and treatment services, including social discrimination, cultural biases, and lack of information and services (9, 29, 30). Our analyses revealed that 34.93% of participants currently maintain steady homosexual relationships, indicating stability and specific partner preferences within MSM community subsets. A consistent HP might signify an enduring and supportive relationship with emotional and sexual stability. These stable partnerships can be crucial in decreasing risky behaviors and the risk of HIV transmission among MSM. Furthermore, maintaining a stable HP can foster positive health behaviors and enhance HIV prevention efforts. Such relationships provide emotional support, psychological well-being, and happiness and reduce risky behaviors and HIV transmission risks, promoting health-conscious practices and HIV prevention within the MSM community (31). Therefore, advocating for and supporting the development of healthy and enduring partnerships within the MSM community can improve individual health outcomes and advance public health initiatives.

As individuals in the MSM community age, they show a tendency to form stable HPs, likely influenced by shifts in social awareness, sex identity, and perception of sexual orientation. Our analyses indicate that MSM recruited online exhibits a 4% increased likelihood of choosing steady HPs for each year of age progression. This finding suggests that age affects partner selection patterns within MSM communities, corresponding to potential changes in emotional needs and relationship stability. Individuals’ expectations and preferences regarding emotional connections may evolve with increasing age; life experiences and social interactions prompt a focus on enduring emotional bonds rather than momentary impulses. Consequently, older MSM tends toward committed homosexual relationships, seeking depth and longevity in their partnerships. Recognizing the diverse preferences and needs of MSM of various ages is crucial for addressing their sexual health and relationship contentment. Cultural disparities in perceptions and values of enduring partnerships may vary and influence MSM partner selection preferences. Older MSM exhibit lower rates of HIV testing, indicating potential shifts in emotional requirements and health practices as individuals age (32). Offering psychological support and counseling services can help the MSM community navigate the psychological challenges and pressures linked to evolving sexual orientation as they age.

Individuals with higher incomes tend to have excellent economic stability and are better prepared to navigate the responsibilities and challenges of long-term relationships (33, 34). Our analyses indicate that MSMs with an average monthly income >6,000 CNY are 47% more likely to opt for stable HPs, suggesting that income is not solely a financial factor but is closely associated with emotional needs and relational patterns. This finding provides a fresh perspective on the determinants of behavioral choices among MSM. A higher income may signify an enhanced quality of life and a more secure future, prompting individuals to prioritize stable emotional connections. Future research on sexual health services should investigate the implications of this correlation to provide a robust foundation for sexual health policies and intervention strategies.

Exploring the effect of health risk perceptions on MSM partner selection behaviors is valued in sexual health services and prevention. Health risk awareness influences MSM partner selection behaviors (35, 36). MSM who are knowledgeable about the HIV status of their HPs are more inclined to choose stable homosexual relationships. This finding emphasizes the pivotal role of health risk perception in partner selection and its influence on partner selection behaviors, leading to discussions on acquiring health information and making emotional relationship choices. Understanding a partner’s infection status is crucial for making health decisions, preventing infections, and motivating individuals to opt for stable partners to mitigate infection risks. Individuals who perceive using enhancers, including Rush Popper, as increasing the risk of HIV infection tend to prefer steady homosexual relationships. Such individuals prioritize health and safety concerns and opt for stable partnerships to reduce risks. This finding emphasizes the significance of health risk awareness in partner selection. Therefore, examining the relationship between health risk perception and partner selection is essential for promoting sexual health and education. Improving health risk awareness through education and promoting interventions with MSM can enhance their health awareness, behavior risk management skills, and knowledge of health risks, including HIV/AIDS, fostering healthy sexual behaviors and lifestyles to mitigate STD risks.

Individuals who self-identify as exclusively homosexual tend to form enduring relationships with their HPs. This inclination may stem from sexual orientation, which influences expectations and criteria for romantic partnerships. Recognizing and embracing one’s sexual orientation can affect one’s emotional needs and relationship preferences. Therefore, further research is warranted to understand how various sexual orientation–related cognitive factors influence partner selection behavior. Such insights can aid in understanding the evolution of emotional needs and social relationship preferences, offering a scientifically grounded framework for sexual health services and support initiatives (37). In addition, our analyses indicated that men who had engaged in anal sex with other men in the previous 6 months showed a preference for stable HPs. This observation emphasizes the potential link between the frequency of sexual activity and partner selection patterns, which are important for elucidating individual sexual behaviors and preferences. Prioritizing stability and health in partner relationships after engaging in anal sex may lead individuals to choose stable partners to mitigate infection risks and sustain healthy relationships. A deeper exploration of how the frequency of sexual behavior affects partner selection could promote healthy sexual choices and behavioral management within the MSM community.

Consistent condom use helps reduce STD risks, especially in the prevention of HIV/AIDS and other infections (38, 39). Our analyses indicate that men who have engaged in anal sex with other men in the previous 6 months and used condoms consistently are less inclined to form stable HPs. The psychological and emotional dynamics of stable partner relationships might influence this behavior. In such relationships, establishing trust and stability leads individuals to view their partners as dependable, potentially decreasing their motivation to use condoms. In addition, communication norms in stable partnerships often facilitate discussions on sexual health, potentially improving awareness of STD risks and lowering the perceived need for condom use (40). Nevertheless, research has shown that stable partner relationships do not eliminate infection risks. Therefore, emphasizing the importance of safe sexual practices, engaging in timely sexual health counseling, and regular testing is critical to safeguard one’s health and that of one’s partners. Promoting sexual health and education should emphasize the necessity of adhering to safe sexual behavior guidelines within stable partner relationships to promote the sexual health of all parties involved.

One-night stands often prioritize immediate sexual gratification and experiences rather than long-term, stable emotional relationships, leading to a lack of enduring emotional investment and intimacy (41). Our analyses indicate that MSMs who participate in one-night stands are less inclined to pursue stable HPs. This observation emphasizes the temporary and independent nature of one-night stands in sexual behavior patterns and partner choices, offering insights into sexual behaviors and emotional relationship decisions. Consequently, individuals within the MSM community who have engaged in one-night stands may lean toward maintaining temporary independence without committing to a fixed HP. One-night stands may lack a deep understanding and trust between partners, potentially increasing the risk of STDs. Therefore, MSM who have experienced one-night stands may tend to refrain from immediately establishing lasting HPs to mitigate possible health and emotional risks (42). These findings elucidate the sexual behavior patterns and partner selection among MSM, facilitating the development of more targeted sexual health intervention strategies.

This study has several limitations. First, the research methods may have bias. This study’s participants consist of MSM aged 18–60, who were recruited via the social media platform WeChat by NGO. Recruitment of MSM through the Internet could introduce selection sample bias since only MSM with Internet access and willingness to participate could join the study, potentially misrepresenting the full MSM population. The use of WeChat may introduce biases toward specific identity groups, as younger and more open-minded participants may be more likely to engage in the study. Therefore, we specify in the title that the participants are MSM recruited through the Internet. Furthermore, certain study variables may have limitations in their definitions and measurements. For example, the definition of a stable HP may vary depending on individual interpretation, causing ambiguity. The definition and measurement of this variable in this study might not have captured the participants’ actual circumstances accurately, potentially affecting the accuracy of the research results. In addition, the information and conclusions of this study may have constraints. The inherent nature of cross-sectional studies limits the exploration of causal relationships between variables and the establishment of causality. Therefore, prospective cohort studies are essential to validate and expand the findings of this study.

## 5 Conclusion

Individuals showing greater preference for stable HPs considered age, income, self-identified sexual orientation, sexual history, and awareness of their partner’s infection status. Engaging in one-night stands and recognizing the importance of consistent condom use during sexual activities diminished the inclination to choose stable HPs. Strategies aimed at preventing HIV transmission among MSM should prioritize interventions and awareness campaigns directed at their sexual partners to guide and enhance their awareness of health risks and protective measures while strengthening sexual education and advocating for safe sexual behaviors.

## Data Availability

The original contributions presented in the study are included in the article/Supplementary material, further inquiries can be directed to the corresponding author.
